# Homœopathy

**Published:** 1890-04

**Authors:** 


### BOOK NOTICES.

HOMŒOPATHY—A FEW THOUGHTS FOR THE INTELLIGENT. By S. Mills Fowler, M. D., Gainesville, Texas.

This is the title of a tiny vest-pocket pamphlet published by Dr. Fowler, for private circulation among his patients. It is a very clever setting forth of the principles of Homœopathy, and very suitable for circulation among the laity. W. M. J.

